# The Post-Normal Challenges of COVID-19: Constructing Effective and Legitimate Responses

**DOI:** 10.1093/scipol/scab037

**Published:** 2021-06-07

**Authors:** Stephen Rainey, Maru Mormina, Sapfo Lignou, Joseph Nguyen, Paula Larsson

**Affiliations:** Oxford Uehiro Centre for Practical Ethics, University of Oxford, Littlegate House, St Ebbes Street, Oxford OX1 1PT, UK; Ethox Centre, University of Oxford, UK; Neurosec, University of Oxford, Oxford, UK; Oxford Uehiro Centre for Practical Ethics, University of Oxford, Littlegate House, St Ebbes Street, Oxford OX1 1PT, UK; Centre for the History of Science, Medicine, and Technology, University of Oxford, UK

**Keywords:** COVID-19, post-normal science, governance, rights

## Abstract

The ongoing COVID-19 emergency clearly presents novel challenges, both in terms of difficulties for maintaining public health and in assuring that governmental responses are ethically sound. Centrally, responses must respect, as best as possible, fundamental human rights and human values. Conflicts among values arise in response to the crisis, and public officials have no choice but to prioritize some while sacrificing others. Utilizing the concepts of effectiveness and legitimacy within the framework of post-normal science (PNS), we investigate and recommend processes and measures to address COVID-19 that support increased public health, while upholding established rights and values. The effectiveness and legitimacy of science-led policymaking requires investigation of how that policy ought to be made (e.g. concepts of policymaking and PNS), as well as how it ought to interact with diversely-constituted publics (e.g. public inclusion in policymaking and policy communication).

## 1 Introduction

Faced with a novel viral pandemic, the state (i.e. government, national agencies, institutions, and citizenry) has to face its challenges in some kind of politically legitimate manner. Policy and governance responses to a pandemic must be proportional in at least two senses: proportionate in terms of effectively tackling the viral threat; proportionate in terms of distributing burdens among the citizens owed protection from the virus. The measures undertaken cannot themselves be too weak, so as to fail to address the viral threats, nor too strong, so as to overburden the targets of the response.

For example, a swift and total shutdown of public life might serve to most effectively stop viral spread, but might also prove to disproportionately hit those from socioeconomically-disadvantaged groups harder than others (Wright et al. 2020). Conversely, where least infringement of liberty guides response, those same disadvantaged groups might still endure disproportionate hardship in having fewer liberties to take (e.g. those working in crowded, public environments will be more consistently exposed to viral threats than others working in private offices) (The Lancet 2020). Relatedly, the threats from the virus are real for all, but those typically most medically at risk tend to be older, while those most likely to experience economic threats as fallout from the pandemic tend to be younger (Mallapaty 2020; OECD 2020). How to disentangle such complexities, especially in a situation with fast-moving information changes, is clearly difficult and perhaps without any ideal outcome (O’Mathúna 2016).

While it becomes necessary to ruminate on the goals of pandemic governance and the proportionality of the means of realizing them, government must nonetheless act quickly. The protective duty of the state towards its citizens requires this. But this duty too must be evaluated in terms of proportionality. For sure, under pandemic governance the goods typically associated with the human rights laid out in the UN’s 1948 declaration will not be fully realisable (‘Universal Declaration of Human Rights’ 2015). Restrictions in the name of ‘lockdown’ will constrain the ways in which certain rights may be realized including:

Article 13: (1) Everyone has the right to freedom of movement and residence within the borders of each state; (2) Everyone has the right to leave any country, including his own, and to return to his country.

Article 20: (1) Everyone has the right to freedom of peaceful assembly and association.

Article 21: (2) Everyone has the right of equal access to public service in his country.

Article 27: (1) Everyone has the right freely to participate in the cultural life of the community…

Such curtailments can be considered justifiable in the name of maintaining adequate standards of living, ensuring public health, protecting rights to life and security, as well as in terms of the individual’s duties to wider society. This is enshrined in articles of human rights, including:

Article 3: Everyone has the right to life, liberty, and security of person.

Article 25: (1) Everyone has the right to a standard of living adequate for the health and well-being of himself and of his family [sic]…

Article 29: (1) Everyone has duties to the community in which alone the free and full development of his personality is possible; (2) In the exercise of his rights and freedoms, everyone shall be subject only to such limitations as are determined by law solely for the purpose of securing due recognition and respect for the rights and freedoms of others and of meeting the just requirements of morality, public order, and the general welfare in a democratic society.

Law can be used to advance health, through making binding commitments on citizens for instance, but may also put pressure upon the provisions of human rights. Individuals’ rights and freedoms can justifiably be constrained where public health may be impacted by their ordinary enjoyment. This includes where a standard medical service is withdrawn, or treatment delayed, in order to address a pandemic threat. This has been seen where medical provisions must reorganize themselves in order to boost capacity when faced with the possibility of collapse through sheer numbers of potential virus patients (e.g. Booth and Campbell 2020). Further impacts relate to considerations of prioritization of active and palliative treatments, and the curtailment of community-based caregiving in the face of COVID-19 risk (Spicer et al. 2020). The tension between rights to health and duties to recognition of lawful requirements for general welfare is high. Especially in addressing this tension, it is essential to carefully consider how expert advice ought to feature in policymaking.

We can investigate these issues through consideration of some key governance concepts, and the idea of ‘post-normal science’ (PNS), which we use here as a normative concept to alert to the existence of high stakes and high uncertainty in the decision-making process and the need for extended knowledge (scientific and non-scientific) to inform policy. PNS requires creating spaces for public deliberation where the scientific process normally confined to scientific experts engages with an extended peer community (EPC) involving all those with a stake in the problem and including different perspectives and values. We see PNS as a key factor not just for the legitimacy of decisions, but for the ‘quality’ of the knowledge that informs those decisions and therefore their effectiveness. We then consider how communication of decision-making is pursued, and how, and to whom, it is addressed. Finally, we reflect upon how governance of emerging crises might be used as an inflection point in order to realize better governance in general, which in this case we present in terms of an equity-based approach to healthcare provision.

## 2 Effectiveness and legitimacy

It is especially apparent, under conditions of emergency, that policy effectiveness and legitimacy can be competing forces. ‘Following the science’ is one vital element for effectiveness where a pandemic is concerned. ‘Following the science’ raises reasonable questions including, which science, and why? In what sense ‘follow’? To what degree? The idea of creating arguments ‘from science’ for any given policy is tempting, for it ‘borrows’ the perceived authority of science to legitimize decisions. In practice, however, it is not guaranteed that any given citizen will share the assumptions or knowledge base of the experts and submit themselves to policy strictures, even if they trust scientists more than politicians. More than careful curation of information and efficient communication of scientific evidence is essential to ensure effectiveness and legitimacy coincide. It is crucial to engage openly and explicitly in a debate on values and principles. While scientific evidence may tell us what works on any given policy scenario, it does not help us determine desirable ends or legitimate means. As David Hume famously stated, it is not possible to derive an ‘ought’ from an ‘is’.

If science is a crucial (albeit not the only) element in policy making, the inescapable epistemological, communication, and ethical aspects of science must also be viewed with increased attention and sense of responsibility. Science is multifaceted and far from univocal, especially so in conditions of high uncertainty`. Advocating for change in social norms on the basis of science alone does not automatically provide the means for its acceptance among the plurality of public actors with different values, interests, and perspectives. ‘Science’ cannot be assumed sufficient to motivate action. The debate over genetically-modified foods (Devos et al. 2008) illustrated this clearly, so too in areas such as nuclear power (Lacassin and Lavelle 2016), and increasingly in vaccination (Hotez 2020), climate change (Björnberg et al. 2017), and the rollout of 5G (Nguyen and Catalan-Matamoros 2020). Scientific advice does not translate into public action just in virtue of the advice being scientific. Historic trends in anti-vaccination sentiment have demonstrated the continued endurance of certain arguments through time, ranging from accusations of scientific tyranny, to blatant refusals to believe the scientific consensus (Porter and Porter 1988; Larsson 2020). Without a construction of the norm in terms of the views of that set of addressees, the conditions for the application of the norm (as opposed to the conditions for its justification) can’t be assumed.

Essential to analysing this situation is understanding policymaking in terms of (1) an instrumental sense of *effectiveness* and (2) *legitimacy* in responsiveness to public rights and values. Where a novel viral threat is present, it must be accepted that knowledge of infectiousness, mortality, transmission modes, and other such dimensions will be incomplete and evolving. Nevertheless, scientific advice must find its way into ongoing (and urgent) policy deliberation. The question is: how can policy decisions made on the basis of incomplete, uncertain, and contested scientific advice retain legitimacy? Does simply conveying decisions and decision-making to the citizenry transparently make it possible for social actors to change their minds in support, or at least recognition, of those decision processes’ validity? We argue that legitimacy and compliance both require justification of and transparency about the choices of science and experts, understanding citizens’ contexts, and explicit engagement with their values. Only with this more complex perspective can ‘following the science’ be made sense of for science, policymaking, and citizen alike. This multidimensional, value-aware approach, which we discuss more fully in the next section, can ensure that policies respond to the lived realities of people and appeal to their values, thus enabling the rational adoption of behavioural changes by citizens and consequently increasing policy effectiveness and legitimacy.

In terms of legitimacy, important questions arise surrounding the necessary restrictions of rights that will emerge in any serious response to a novel viral pandemic. Reprioritizing human rights implementation will be required where viral spread is to be controlled through diminution of the normal scope for liberty, for instance. COVID-19 specifically has clearly presented exceptional circumstances, under which the need for rights curbs has emerged. The Siracusa principles are widely known, internationally agreed-upon, bases for restricting human rights under exceptional circumstances (Debevoise and Lindsay 1985). The principles allow for rights restrictions, provided that the restrictions are:

- In accord with law.
- In the interest of a legitimate objective.
- Necessary to achieve the objective.
- The least intrusive and restrictive means available.
- Based on scientific evidence.
- Not drafted or imposed arbitrarily.

Among these desiderata for permissible rights restrictions are that such restrictions be, ‘The least intrusive and restrictive means available’, and ‘Based on scientific evidence’. But these conditions themselves require considerable interpretation. For instance, ‘least intrusive’ might also be least effective, in failing to contain viral spread. Besides, it can be asked, ‘least restrictive’ for whom: restricting freedom of movement will impact more upon those with less private room in which to constrain themselves. Apart from these, the idea of restrictions ‘based on science’ raises questions like, *how* and *which ‘science’?* Ought policy to respond to behavioural scientists, epidemiologists, virologists, modellers, historians? And having curated a scientific knowledge bank, ought policy to be determined by that bank, forfeiting political control, or merely steered by it, retaining power?

The Siracusa principles are clearly a useful means of considering rights restrictions as legitimate, but they conceptualize legitimacy in procedural terms. This could reduce ‘legitimacy’ to simply conveying that the person announcing the policy is appropriately empowered within their organization. ‘Legitimate policy’ can be taken to mean, ‘policy arrived at by appropriate channels’. Somewhere among these and other interpretations, the legitimacy of policy must be investigated, alongside its effectiveness. This can be achieved through reflection upon the manner in which normative injunctions are contextualized for specific citizens and groups. This minimally involves distinguishing and communicating the different justifications for norm changes (the content of policy), and for the acceptance of norms (the context of policy reception). These open questions for legitimacy along a spectrum from mere institutional logic, and procedurally correct creation of policy, to the complexities of responsible agency, and the rational adoption of norms. The sense of legitimacy attached to this latter sense of responsible agency is as important but requires completely different consideration relating to ethical accounting for value, rather than responsibility to institutional logic (Ayres et al. 2017; Šucha and Dewar 2020).

Taking the example of the UK, the response to the virus can be seen as ‘legitimate’ in the institutional logic sense of following established law and procedures. Public Health England (PHE), sponsored by the UK’s Department of Health and Social Care, provided national and local government, the National Health Service (NHS), Parliament, industry and the public with evidence-based professional, scientific expertise, and support. In the context of an emergency like COVID-19, PHE and the NHS use an incident governance and organizing model that is promoted by the Prime Minister’s Cabinet Office, tasked with aiding overall delivery of national government. Their model is widely used across the UK and is based on an emergency response model laid down in statute, namely the Civil Contingencies Act 2004. Each responding organization uses an incident response structure akin to project management in that it operates like a temporary organization but structured to address immediate operational needs, seen nonetheless in a wider context of strategic future goals. In this sense, it is similar to a military approach with immediate goals as presented by circumstance but within overall terms of engagement. Alongside the procedural legitimacy of its response, the government sought to enact its commitment to effectiveness of policy by convening independent scientific advice for policymaking via the UK Scientific Advisory Group for Emergencies (SAGE) (‘Scientific Advisory Group for Emergencies’ n.d.).

The weight of scientific advice in UK policy decisions was evident in sloganized messages which, at the height of the pandemic, sought to reassure the public that the government was ‘following the science’. Short of an empirical study about the specific processes of science informing policy during the COVID-19 pandemic, we can nonetheless refer to one recent past example to appreciate the dynamics at the science-policy interface. The 2009 Swine flu epidemic in the UK revealed dislocations between local and national policy response approaches which, as Chambers et al. (2012) argue, were compounded by, ‘… over reliance on the speculative logic of modellers, together with a failure to adapt swiftly the nation’s preparedness plans and public health apparatus created in readiness for a serious and fatal disease’ (2012: 737) which opened questions about what constituted scientifically acceptable and politically legitimate advice. The UK’s decision to centralize response to swine flu comes in for particular criticism:

The imposition of a single national approach to managing the pandemic and a disregard for the role of local authorities seriously impaired the ability of local agencies to respond in a ﬂexible, timely and pragmatic way to the rapidly emerging situation. (2012: Loc cit)

Similar notes of criticism of COVID response have been noted since March 2020 (Health Foundation 2020). In particular, serious concerns were raised by the muddying of scientific ‘leadership’ versus ‘guidance’ in policy, as well as the inclusion of very influential government advisors within supposedly independent scientific panels (Torjesen 2020). And local responses have been stalled in favour of centralized approaches (Dyer 2020).

We are not problematizing scientific practice but rather the way in which it is embedded in the policy process, in particular the blurred boundaries between scientific advice and political accountability and the perceived opacity and lack of impartiality of the advisory process that risk undermining public trust. The inclusion of scientific insight is necessary to increase policy effectiveness (though more evidence does not always mean better policies) but it must also be legitimate. However, effectiveness and legitimacy can be seen in tension, and ultimately undermined, as the swine flu precedent demonstrates. In the context of COVID-19, reliance on scientific insight was necessary to counter a novel threat but to also to legitimate political actions, especially those aimed at curtailing individuals’ democratic rights and freedoms. The contentious ‘following the science’ slogan sought to appeal to the authority of science as the ultimate arbiter of decisions. However, effectiveness was compromised by government actions that departed from scientific advice to prioritize political and economic interests (Ahmed 2020a; Torjesen 2020), while legitimacy was called into question as the political independence and representativeness of SAGE became increasingly scrutinised (Bacevic 2020).

Intense criticism of SAGE’s secret membership (‘COVID, transparency and trust’ n.d.) and its suspected of lack of diversity and disciplinary representation raised questions as to ‘what’ science the government was following, ‘whose’ knowledge counts and more importantly, ‘whose’ is being excluded (Mormina et al. 2021). This ultimately led to the establishment of organized scientific opposition in the form of a group self-referred to as ‘Independent SAGE’ (Horton 2020) that further undermined the legitimacy of the official scientific advice. This contradicts the slogan’s implicit presumption that there was one ‘science’ to follow. Science did not, and does not, speak with one voice: over the course of the pandemic scientists aired their disagreements, often ferociously, in the public arena, evidence was contested, advice reversed. In other words, the immutable facts of science have been constantly reconstructed and reconfigured as they passed through the prism of individuals’ perceptions and understandings, whereas scientific certitudes fell apart as they entered a new space where ‘facts are uncertain, values in dispute, stakes high and decisions urgent’ (Funtowicz and Ravetz 2020). It is precisely because of this entanglement of science, ethics, and policy, where the latter needs to (urgently) bridge the (uncertain) ‘is’ of the first and the (disputed) ‘ought’ of the second, that it is crucial to ask not just what science guides decisions but crucially ‘who’ are its heralds. Such concerns are all the more central as vaccination programmes are rolled out nation-wide amidst substantial increases in vaccine hesitancy (Luyten et al. 2019).

In sum, the legitimacy of a scientifically-informed policy cannot come solely from its effectiveness as based in a science that is internally contested and externally questioned for its independence, nor from this in combination with a correct pursuit of procedures. Procedure and (scientific) authority are necessary but not sufficient conditions for legitimacy. In addition to these, legitimacy must be derived from some account of the good. In other words, a policy is legitimate because it is somehow more generally good, and perceived to be good by those affected by it. This moves us out of the space of facts and into the realm of ethics and values to define what counts as a good set of outcomes. That is, it moves us into the space of ‘PNS,’ and its constituent focus on science, policy, and the public.

## 3 Pandemics as ‘wicked problems’ and the policy response

Pandemics are a typical example of what Rittel and Webber (1973) famously conceptualized as ‘wicked problems’. Unlike mechanical, linear, or ‘tame’ problems for which both causes and solutions are knowable, complex ‘wicked’ problems present a less linear and more reticulate pattern of social, biological, and economic relations for which explanations are less certain and quantitative predictions more speculative and unreliable. It is in this context of limited informational certainty, compounded by time pressures and high stakes, that critical decisions need to be made. Where should limited supplies of personal protective equipment (PPE) go? Who should be vaccinated first? Which scientific model should be trusted? When should children go back to school? Where does the balance lie between saving lives and protecting livelihoods? These are decisions that cannot be arrived at by only plotting a line on a graph. They require hard moral (and political) discernment.

A particular feature of wicked problems is that they lack definition because multiple and often incompatible characterizations are possible depending on the agents’ perspectives and underlying values. Since there is no single definition of the problem there can be no single answer but a variety of multiple and often contradictory solutions. This was particularly evident during the early phase of the pandemic in 2020 when uncertainty regarding the features of the virus divided opinion between those for whom the outbreak was similar to the seasonal flu and those who regarded it as a novel pathogen (Chen et al. 2020; Rettner n.d.). For the latter, the only option was to focus on suppression strategies (acknowledging the unknown nature of the virus and prioritizing the medical perspective and the value of preserving human life), while the former preferred mitigations strategies based on the pursuit of herd immunity (favouring flu-like comparisons and prioritizing the economy) (‘Declines in COVID-19 cases not due to herd immunity, says analysis | Imperial News | Imperial College London’ n.d.; Titheradge and Kirkland 2020). Another feature of wicked problems is that they are not a single problem but a mesh of multiple interrelated problems with complex and tangled roots. They must be tackled together or cannot be tackled at all. Addressing one aspect of a wicked problem will create or exacerbate other aspects. For example, public health measures aimed at containing the spread of the disease by limiting social contacts may suppress the virus, but in the process may also compromise other health outcomes related to people’s capacity to sustain livelihoods, their mental health, or access to other essential medical services.

The paradox of wicked problems is that they are by definition unsolvable and yet demand action. One way of resolving this paradox is by turning a wicked problem into a ‘tame’ one, that is, reframing it so that it becomes a bounded problem amenable to a solution or set of solutions (Daviter 2017). Scientific advice may be sought to help reframe the problem and/or identify solutions, sometimes via technical independent agencies (such as PHE or its USA equivalent, the Centre for Disease Control or CDC), or *ad hoc* epistemic communities (such as SAGE). These ‘boundaries organizations’ (Guston 2001) work at the interface of science, policy, and the public but often with a rather specific mandate of risk assessment involving systematic, analytical, and largely probabilistic information gathering to characterize threats, model its distribution, estimate its risk, and communicate it to society and policy makers Timotijevic et al. 2013). Given these narrow terms of reference, members are appointed to these organizations on the basis of their technical expertise and mostly recruited from a narrow set of so-called ‘hard sciences’, consistent with a technocratic model of policy making that rests upon a Kuhnian view of ‘normal science’ as puzzle solving (Kuhn 1996).

Tasking these boundaries organizations to tame a wicked problem by reframing it may be an appealing approach that allows swift action and the clear assignment of responsibilities to a small group of technical experts, but it narrows the set of available expertise, limits debate and participation, and thus casts aside competing perspectives (Daviter 2017). A clear example of this in the COVID-19 situation has been the—arguably uncritical—overreliance on statistical models. More than with previous epidemics, statistical modelling has been the dominant form of expert advice to governments. Models can emerge as the only source of information when empirical data are scarce or missing, such as is the case with a novel virus, but they are as good as the assumptions fed into them. The influential model from the team at Imperial College London (Ahmed 2020b) which weighed heavily into the UK government decisions in early March, was not only based on an old code for influenza pandemic and underestimated demand for intensive care units, but crucially did not consider the impact of rapid testing, test, trace, and isolation, key components of the public health toolkit (Sample 2020). Had diverse perspectives been consulted, key insights from public health, or social science could have provided essential challenges to the model’s assumptions. Involving the public in the introduction of modelling too could provide important perspectives that a statistical model cannot obviously capture, even if it relies on correct assumptions.

## 4 Post-normal science

Pandemics, like wicked problems, are characterized by informational uncertainty and system complexity. They expose the limits of normal science and call for what Funtowicz and Ravetz call ‘PNS’ (Funtowicz and Ravetz 1993). The concept, which was originally developed as a methodological corrective to the shortcomings of normal science operating in contexts of urgency, uncertainty, disputed values, and high stakes, incorporates ethical and social considerations as variables, alongside scientific facts, in the management of complex problems. In their iconic representation of PNS (ibid: 750), Funtowicz and Ravetz described complexity along two axes: decision stakes (potential costs and benefits to relevant parties) and system uncertainties (technical, scientific or managerial aspects, and their outcomes). When decision stakes or system uncertainties are high, there are no precedents for managing the issue and no agreed scientific or technical solution, yet something must be done. In these situations, finding a way forward to manage uncertainty requires stepping outside the boundaries of science and engaging with questions of value, ethics, and politics. It is therefore necessary to create new epistemic structures, EPCs, where scientific knowledge enters in dialogue with other relevant kinds of knowledge. In contrast to scientific knowledge, which can for instance be communicated statistically, other relevant kinds of knowledge (e.g. specific practical knowledge due to social roles) can be expressed through participation in social action. EPCs are constituted by all those with a stake on the issue: policy makers, communities, and individuals, each contributing their ‘extended facts’ (local knowledge, contextual understanding, etc.) that may not be available to scientific experts, and without which normative issues cannot be addressed (Turnpenny et al. 2011). Besides the contribution of extended facts to the knowledge base required for decision making, EPC bring also a quality control function (Funtowicz and Ravetz 1997) by acting as an extended peer-review board that evaluates the epistemic quality of the decision (i.e. the completeness of the knowledge upon which that decision is based, as assessed by the spread of epistemological, cultural, and moral perspectives included). While ‘normal science’ focuses on truth understood as the objective representation of reality, PNS focuses on quality, understood as enhanced participation and epistemic inclusion.

While originally conceived as a methodology to address the inability of traditional science to deal with wicked problems (and thereby to increase its effectiveness), PNS intersects with issues of trust and legitimacy in decision making by opening up space for disagreement and presenting opportunities to reach collective conclusions. It therefore is fundamentally normative. It is in this latter sense that we engage with the concept. Indeed, despite the ‘science’ in its name PNS is less about scientific knowledge production and more about the politics of decision making (Wesselink and Hoppe 2011). Like Funtowicz and Ravetz, we understand PNS as a space for democratic deliberation where the EPC comes together to contribute extended knowledge to the decision-making process and exert a quality control function on that knowledge. For example, the development of new methodologies for co-production of knowledge was successfully done by Lowrie and Tyrrell-Smith in their work identifying priorities in child health in a socioeconomically-disadvantaged community in Blackpool (Lowrie and Tyrrell-Smith 2017). Such methodologies can be central to the effectiveness of the decision. Considering it as an effective process to collect and generate relevant information for effective decision-making, we see PNS as fulfilling also a legitimizing function, where the deliberative process within the EPC (provided it represents all those with a stake in the problem) allows for the exploration of a range of views may result in consensus or consensual compromise—and therefore legitimacy—about the decision arrived at.

With the PNS notion of ‘quality’ on the table, it can be seen how effectiveness and legitimacy are inter-related. Effectiveness will require legitimacy just as legitimacy will require effectiveness. This adds urgency to the question of how to approach constructing the problems of the COVID-19 emergency, as well as identifying the publics who will be the addressees of the solution. For example, it must be asked whether COVID-19 is most importantly a public health, or economic problem? Depending upon how this question is approached, and answered, further questions arise for coming to a solution, such as *which experts to consult*? In terms of PNS, this amounts to trying to predict who forms the EPC: how are they selected, for what ends, and whose voices are key?

## 5 Practical considerations

Balancing the needs for inclusive, yet swift action is the ideal of pandemic response. In practice, however, the ‘wickedness’ and urgency of a pandemic does not always allow for inclusive approaches. Complexity and lack of agreed definitions mean that different actors will see/prioritize a specific aspect of the problem, leading to potentially complex and dynamic disagreements. Without unequivocally defining the problem, an adequate solution cannot be grasped either. Thus, for practicality’s sake, a common strategy for dealing with wicked problems is to limit deliberation (either by narrowing its scope, constraining participation, or both) so as to reduce the potentially-disruptive role disagreements might play, and thereby facilitate quick decision making. As we outlined above, this is a strategy that neither boost effectiveness (though it may aid urgent decision-making) nor is it satisfactory from a legitimacy point of view. By overtly limiting input deemed problematic so as to aid consensus-building, the likely realities of reasonable dissensus are excluded and the risks of groupthink increased. Dissensus may be more legitimate (and effective) a basis for difficult decision-making than an artificial consensus constructed in the rush to act (Wilkinson and Savulescu 2018). Especially where an EPC is considered central to a legitimate strategy for conceptualizing a problem for which a solution is sought (as it is here), complexity must be acknowledged among the plurality of those for whom the problem is a problem. This entails a reflection upon the construction of the EPC and the challenges of respecting and responding to a variety of views, values, vulnerabilities, and stakes.

General reflection on how governance decisions regarding pandemic responses impact the lives of people with different health, social and economic needs, and risk profiles highlights the need for extended knowledge. By expanding the informational basis through consultation and engagement through EPC, it is possible to base decisions upon evidence that better reflects the diversity of realities experienced by all citizens and the societal impact of policies. The democratization of science through co-production of knowledge within an EPC can lead to higher quality decision-making (in the PNS sense elaborated above). For policy decisions to be more effective and legitimate, and to be informed by the reality and concerns of different communities, opportunities for inclusive public involvement are essential. In practice, however, any attempt to democratize science requires a much deeper engagement with the role of power relationships than PNS theorists acknowledge.

Participation in an EPC is not as egalitarian as Funtowicz and Ravetz appear to suggest (Wesselink and Hoppe 2011; Karpińska 2018) and different voices have different power. Consider for example the influence that behavioural scientists and modellers have had on government decisions over traditional public health advice (Hunter 2020), an approach reminiscent of the UK’s Foot-and-Mouth Disease outbreak in 2001, when the models of a particularly influential group of scientists (equally influential in the current COVID-19 pandemic) drove government response, dominating over the knowledge of farmers, vets, and countryside dwellers, with detrimental consequences (Kitching et al. 2006). EPC can be an instrument of exclusion when, for example, it is used to further the interests of particular lobby groups (Timotijevic et al. 2013). This can undermine what is at the heart of a deliberative model—rational discussion between free and equal citizens. The exclusion of certain people’s perspectives and kinds of knowledge in deliberation raises issues of procedural fairness. This presents particular problems when excluded voices are of those who have faced marginalization or oppression in the past. In this regard, power becomes an important variable to understand not just how EPC are constituted and who participates in them but also on what terms.

As Wesselink and Hoppe (2011) point out in their critique, ‘inviting less privileged groups to the table is not enough to change policy direction’. Participation is a social process, and like all social processes it is determined by hierarchies, lobbies, and relationships. Funtowicz (2006) is probably aware of this when he insists that PNS is above all about dialogue and the ‘quality control’ function of the scientific evidence, not about the policy decision. However, trying to separate the two is difficult. Moreover, it dodges the question of how to distribute power beyond the ‘usual suspects’ and avoid lip-service enagagement within EPC. One option is to control stakeholder interaction by adopting a structured approach through narrow framing. This approach may avoid agenda capture by the aforementioned usual suspects, as well as provide a focal point for dialogue, but it restricts the range of views that can be expressed. In the context of a rapidly evolving pandemic, it is difficult to assess the relative influence of different voices at this time. A small example in the UK may be the successful campaign for providing social security top-ups to low-income families led by a high-profile footballer, which brought a drammatic policy u-turn and put the issue of child poverty high on the government agenda (Bowkett 2020). Although in this case the campaign attracted huge support from all sides of the political spectrum, it nonetheless exemplifies the disproportionate power of certain voices to advance their causes. Future research may shed light on how power dynamics within the various formal and informal epistemic communities influencing policy during this time may have drawn and redrawn the pandemic response path of governments around the world.

Involving citizens in the decision-making process is important not only to inform policy decisions on what works (effectiveness), but also on what matters (values) (Lavelle and Rainey 2013). Triage decisions, for instance, raise moral questions of how to balance individual rights and collective interests and risks, and whose needs should be prioritized. These questions concern societal values and must be subject to carefully constructed deliberation. It is through deliberation on values that a shared account of the good can be found. This is not just important to legitimize policy decisions, as we argued earlier, but especially to frame problems and set agendas in the first place. A major criticism of PNS as a methodology to address wicked problems is that it is often a deliberative process that happens within the narratives (agendas) framed and controlled by small political elites and does not sufficiently challenge their hegemony (Karpińska 2018). This undermines deliberation as a process of social learning. Echoing this critique, Wesselink and Hoppe (2011) argue that PNS should start with agenda setting, that is, with deliberation on problem definitions and framings predicated on an engagement with values.

Our analysis of the events of 2020, in the UK and other countries, leads us to concur on the need for meaningful processes of inclusive deliberation that are not constrained by pre-defined framings and agendas controlled by political elites. Notwithstanding the cacophony of voices, commentary, and analysis, the public discourse around Covid-19 has taken place, not within a broad PNS space but within narrow and politically-charged narratives; as a consequence, it has been impoverished. For example, many policy choices were framed, and therefore debated, as a ‘trade-off’ between protecting lives and protecting the economy. This framing has been exploited by both sides of the political spectrum and has exacerbated social polarization in the UK and USA. In these national contexts, the political left (in opposition) emphasized the paternalistic role of the state in safeguarding health and wellbeing and a communitarian vision of society, whereas the right (in government) focused on a vision of autonomous, atomized individuals, and guarded against an overly intrusive government encroaching on citizens’ liberty. This framing not only has reduced science advice to a mere risk assessment of the trade-offs between the goals of public health and the needs of the public purse, but more importantly it has obscured deeper societal questions, such as what ought to be the right role of the state (e.g. paternalistic or laissez-faire) or the principles for a good society (e.g. liberalism or communitarianism). While we acknowledge the challenges of applying PNS to public policy, as discussed above, we can speculate on what a deliberative process may have looked like if more open to an early engagement with PNS approaches at the stage of problem formulation and less bounded by political narratives, as Wesselink and Hoppe (2011) recommend. Such early engagement may have perhaps arrived at a less polarizing framing of the pandemic that does not pit the health of the nation against the health of the economy but sees them as mutually dependent, resulting in a very different scientific and policy response in these countries.

For this reason, it is important to design effective forms of public deliberation and participation that seek to build consensus around a shared account of ‘the good’ in order to avoid increasing polarization. Discussions should reflect the spectrum of views and concerns and not be framed by dominant groups. Open deliberation should not conceal institutionalized inequality but encourage diverse citizen voices by including a variety of stakeholders in the process: charitable bodies, community groups, civil society groups, and others. Public engagement must be based on clear communication and fair procedures to produce legitimate decisions and effective outcomes (Hajdu and Simoneau 2020; Sienkiewicz et al. 2020). Inclusive deliberative processes must not be seen in opposition to representative democracy and those with political power must be ready to cede power and ensure that citizens have forums in which governance is shared and voices are not excluded. In practice, this requires overcoming vested interests and creating, from the bottom up, demand for reimagining the existing processes, structures, and institutions, and the rules and cultures shaped by them, and within which PNS-type approaches to this pandemic or indeed any other wicked problem remain only aspirational.

The pandemic above all has brought into relief various conflicts among ‘values’: responsibility and solidarity versus freedom and autonomy, stability and welfare versus self-sufficiency, social ties and belonging versus individual independence, wealth and growth versus ecological equilibrium. We do not see PNS as a tool for disseminating scientific information to the public. We see it as a deliberative process that alerts to the existence of uncertainty, and therefore to the need for extended knowledge and a narrative that brings together the plurality of values and unites society around a common goal. To this end, accurate information is particularly important. Public deliberation can be exploited by certain groups to spread malicious information. Therefore, it is imperative that, alongside deliberative processes, there is a comprehensive strategy for combating predicted misinformation and disinformation campaigns. Historical and contemporary evidence demonstrates that times of crisis produce opportunities for significant spread of conspiracy theories and provide fertile soil for misinformation campaigns targeting at-risk and vulnerable populations. False information can be both incidental due to a lack of clarity from governmental sources, or deliberately misleading, with the goal of causing harm. To combat this, pre-emptive misinformation inoculation strategies must be a key aspect of pandemic response communication.

Finally, in arguing for PNS approaches as necessary conditions not just for legitimacy (consensus around some account of the good) but also for the efficacy (quality control fuction) of policy decisions, we recognize that tensions must be reconciled between generalizable (scientific) knowledge and contextual (non-scientific) knowledge. In their analysis of the Netherlands Environmental Assessment Agency (PBL) and their attempts to use PNS methodology to develop national long-term urban development policies, Petersen et al. (2010) provide rich insights into the Agency’s challenges to make use of contextual information to produce knowledge for national-level policy that is independent from particular local contexts. This lesson may be particularly relevant for post-pandemic governance as discussed below, which will require policy makers to generalize the lessons of 2020 to renew and strengthen the social fabric and enhance the national and international preparedness agenda. While contextual knowledge is essential to gain insights into, e.g. the feasibility of policies, it can also undermine the credibility of science (according to the expectation of ‘normal science’) in the policy domain. It is, after all the epistemic authority of traditional Kuhnian science and not PNS that governments say they are following. Until policy makers recognize and demand socially robust knowledge (Nowotny 2003) in addition to scientifically sound knowledge, PNS may remain a normative prescription without political teeth.

## 6 Lessons for post-pandemic governance

In the transition from an emergency state to a post-Covid-19 context, it will be vital to consider the legitimacy of how socio-political decisions are made, especially in terms of how differing individuals and groups have been affected by the emergency and subsequent reflections on public goods, and social disruption. Owing to these kinds of considerations, governments ought to commit to ‘building back better’ and preventing discrimination and inequity persisting rather than singularly focusing on returning to ‘business as usual’ when the pandemic is over. This should include a conscious decision to incorporate collaborative EPC-informed policies. Currently, the focus is rightly on the emergency public health response, but eventually governments will need to learn lessons from the crisis and figure out how to prevent a future recurrence of these challenges (United Nations 2020).

The United Nations in 2015, under the banner of ‘leaving no-one behind’, developed a transformative and ambitious agenda by agreeing to pursue seventeen Sustainable Development Goals (SDGs) (United Nations 2019). It is important to recognize that health equity is only partly linked with the health care system, but extends to the conditions in which one lives, grows, plays, works, and ages, including educational opportunities, meaningful employment, and sustainable places of residence. Indeed, the WHO Commission on Social Determinants of Health defined health equity as the absence of inequalities of health that are reasonably avoidable, and so action on the SDGs are likely to impact health equity directly or indirectly (CSDH 2008). In particular, three goals stand out in relation to health equity:

Goal 3: Ensure healthy lives and promote well-being for all at all ages.

Goal 10: Reduce inequality within and among countries.

Goal 17: Strengthen the means of implementation and revitalize the global partnership.

Inequities are not inevitable and solving them will prove crucial to acceptability and trust in government and the healthcare system. Moreover, health equity is an important indicator of societal wellbeing. Unequal societies are less healthy overall, and this affects everyone within them. Equity should not solely be about uplifting the poorest but uplifting the entire gradient of disadvantage. Individuals have faced the virus from uneven starting points and the effects of COVID-19 has amplified these gaps (de Lusignan et al. 2020). This was especially seen in the heightened infection and mortality risk among minorities, even in economically developed countries like the USA and the UK (Mikolai et al. 2020; Platt and Warwick 2020; Yancy 2020). The devastating impact of some political and economic considerations, such as years of austerity policies in the UK, has also come into the forefront as a driver for widespread inequality.

The 2020 COVID-19 Marmot review showed substantial inequalities in the impact of the pandemic in the UK (Marmot 2020). For instance, difficulty in infection prevention and worsening of existing respiratory health conditions in people experiencing homelessness are factors that make it more likely that an individual develops more serious COVID-19 symptoms (Lewer et al. 2020). The effects of the prolonged health impacts from COVID-19, dubbed, ‘long-COVID’ are likely to have been magnified by those in deprived neighbourhoods, given their increased likelihood of having chronic disease, Overall, differences in mortality rate from COVID-19 relating to deprivation mirror those disparities seen in all-cause mortality rates (England 2020), suggesting that the drivers of COVID-19 inequality are closely related to the social determinants of health, i.e. early childhood, education, employment, housing, and environment. Gains in life expectancy stagnated even before the pandemic, worsening structural inequality, with boys born in the most deprived areas of England expecting 18.9 fewer years of healthy life expectancy than those born in the least deprived areas (ONS 2020). Chronic underinvestment in health systems have meant facilities were fragmented and ill-equipped to cope with the surge in demand brought on by COVID-19.

Government investment in protecting social and economic rights builds resilience and trust. Trust often involves a subjective expectation of what a government *ought* to be doing, and is informed by perceptions of impartiality, relative service provision and justice (Mcloughlin 2015), rather than objective performance metrics. Trust is strongly linked to the legitimacy of a state which can be associated with willingness to abide by the rule of law. To rebuild better, a comprehensive government approach must be taken to develop strategies to combat inequality. The 2020 Marmot Review proposed a strong *whole of society* focus: alleviating childhood poverty, funding education, improving working conditions and minimum wages, and creating the right conditions for people to be healthy (‘Health Equity in England: The Marmot Review 10 Years On’ n.d.). The application of a PNS approach in future policy would enable a fuller realization of this vision. In the context of the COVID-19 response, recognition of the far-reaching impacts of the pandemic and the subsequent response is critical (Berger et al. 2020). For example, a failure to include a community engagement plan may have undermined the awareness of priority populations such as people with disabilities, the elderly and people with mental health illness, who often bear a greater burden of lockdown and quarantine measures imposed (Rajan et al. 2020).

It is clear that a return to the old normal of regressive and deeply unequal society that led to widespread inequality before and during the pandemic is not tenable. But nor is it inevitable. COVID-19 presents current governments with diverging roads: they must step up and choose the path that addresses the drivers of inequities to a healthier, more resilient, and fairer society. For this to be possible inclusive processes of policymaking, like those enabled by an approach embedded within PNS, are imperative to harness a plurality of voices, experiences, and knowledge beyond those already amplified by privilege and power, and to avoid narrow understandings of what justice demands.

## 7 Conclusion

This paper has argued that pursuing the twin goals of effective and legitimate COVID-19 responses involves thinking carefully about the nature of scientific advice and the varieties of people upon whom such advice impacts via policymaking. A threat like that posed by Covid-19 must be tackled *effectively* through carefully adopting scientific advice, but also *legitimately*, so that scientific advice does not dominate political legitimacy.

Adapting scientific advice to policymaking ought to include the public, for reasons of effectiveness in uptake of policy, and legitimacy in responding to public views. This relates directly to policy effectiveness (to address the virus) and legitimacy (to not unfairly impact on citizens). Addressing effectiveness and legitimacy of science-led policymaking requires investigation of how that policy ought to be made (concepts of policymaking, PNS), as well as how it ought to interact with diversely-constituted publics (e.g. public inclusion in policymaking, policy communication). Lastly, in recognizing the rifts laid bare through the various impacts of the pandemic opportunities arise to correct systemic and historic issues relating to inequality and inequity. In addressing these, (1) a human rights-based response more sure-footed and (2) a more resilient policy environment is seeded for future emergencies.
